# Adjuvant Strategies for Lactic Acid Bacterial Mucosal Vaccines

**DOI:** 10.3390/vaccines7040150

**Published:** 2019-10-16

**Authors:** Allison C. Vilander, Gregg A. Dean

**Affiliations:** Department of Microbiology, Immunology, and Pathology, College of Veterinary Medicine and Biomedical Sciences, Colorado State University, Fort Collins, CO 80523, USA

**Keywords:** lactic acid bacteria, mucosal vaccine, adjuvant

## Abstract

Lactic acid bacteria (LAB) are Gram-positive, acid-tolerant bacteria that have long been used in food fermentation and are generally recognized as safe (GRAS). LAB are a part of a normal microbiome and act as probiotics, improving the gastrointestinal microbiome and health when consumed. An increasing body of research has shown the importance of the microbiome on both mucosal immune heath and immune response to pathogens and oral vaccines. Currently, there are few approved mucosal vaccines, and most are attenuated viruses or bacteria, which necessitates cold chain, carries the risk of reversion to virulence, and can have limited efficacy in individuals with poor mucosal health. On account of these limitations, new types of mucosal vaccine vectors are necessary. There has been increasing interest and success in developing recombinant LAB as next generation mucosal vaccine vectors due to their natural acid and bile resistance, stability at room temperature, endogenous activation of innate and adaptive immune responses, and the development of molecular techniques that allow for manipulation of their genomes. To enhance the immunogenicity of these LAB vaccines, numerous adjuvant strategies have been successfully employed. Here, we review these adjuvant strategies and their mechanisms of action which include: Toll-like receptor ligands, secretion of bacterial toxins, secretion of cytokines, direct delivery to antigen presenting cells, and enterocyte targeting. The ability to increase the immune response to LAB vaccines gives them the potential to be powerful mucosal vaccine vectors against mucosal pathogens.

## 1. Introduction

Lactic acid bacteria (LAB) are Gram-positive acid-tolerant bacteria that have long been used in food fermentation and are generally recognized as safe (GRAS). Additionally, they have been identified as probiotics, live organisms that improve health when consumed [1]. LAB are a diverse group of bacteria that includes the genera *Lactobacillus* spp., *Lactococcus* spp., and *Streptococcus* spp. The effects of LAB on mucosal health are diverse and have been most heavily studied in the gastrointestinal (GI) tract. General effects of LAB in the intestinal tract are known to include alteration of the intestinal microbiome composition, improved barrier function, niche competition with pathogens, and, germane to this review, modulation of the host immune system [2,3].

Most pathogens enter the body at mucosal sites and protection of these barrier tissues is mediated by innate and adaptive immune responses. Mucus, peristalsis, gastric acid, bile, and antimicrobial peptides are examples of innate mucosal immune defense strategies while antigen-specific antibodies and cell-mediated responses are the workhorses of the adaptive response. Induction of both innate and adaptive mucosal immune responses is best achieved by direct immunization at the mucosa rather than through systemic routes (parenteral injection) [4,5]. Mucosal vaccines can also induce serum antibody and systemic cell-mediated responses. Mucosal delivery is an especially attractive mechanism of vaccination due to the ease of administration and the common-mucosal immune system, which allows for induction of immune responses at one mucosal surface followed by trafficking of immune cells to other distant mucosal sites [4].

Despite the inherent benefits of mucosal vaccines, there are few available for use worldwide. Of the currently licensed human mucosal delivered vaccines, all are live attenuated or inactivated viruses or bacteria. While these vaccines are effective at stimulating a strong mucosal immune response, the use of attenuated vaccines carries the risk of reversion to virulence and they cannot be used in immunologically sensitive populations [6]. In addition, these mucosal vaccines can have varying efficacy depending on an individual’s health, nutritional status, and microbiome [7,8]. Co-delivery of LAB with oral vaccines has shown the ability to increase the immune response in the face of low nutritional status or dysbiosis. For example, increased immune responses have been seen when probiotics are administered with oral rotavirus, polio, *Salmonella typhi*, and cholera vaccines [9].

Due to the limitations of the currently available mucosal vaccines and the benefits of probiotics on immune response to vaccination, development of LAB as mucosal vaccine vectors is attractive. LAB have several attributes as orally delivered mucosal vaccines including: Acid and bile resistance, stability at room temperature, endogenous activation of innate and adaptive immune responses, and the availability of molecular techniques for genomic modification [10]. Since the 1990s, the use of LAB as an oral vaccine platform has been explored against numerous viral and bacterial pathogens and toxins [11,12]. These vaccines have been shown to induce serum IgG and mucosal secretory (sIgA) as well as stimulate T cell responses. In addition to developing LAB for the delivery of antigens, numerous adjuvant strategies have been explored to enhance immune responses.

Adjuvants are used in conjunction with vaccines to increase the humoral and/or cellular response to a delivered antigen. Pairing the correct antigen and adjuvant can induce faster, more robust, and longer-lived (durable) immune responses, and may decrease the amount of antigen needed to induce protection [13]. Adjuvants such as Alum, MF59, AS03, AF03, virosomes, and heat labile enterotoxin (LT) have long been used with systemic vaccines but adjuvant use has been more limited with mucosal vaccines. Only the intranasal influenza vaccine, Nasalflu, has been licensed for use with a mucosal adjuvant, *Escherichia coli* heat-labile toxin (LT), but it has since been removed from the market [14].

To realize the potential of LAB as mucosal vaccine vectors, an understanding of how to enhance the immunogenicity of these vaccines while preserving the inherent safety will be required. It is likely that despite the endogenous immune activating properties of LAB, one or multiple adjuvant strategies may be necessary to induce robust and long lasting protective immune responses. This may be especially true if the vaccine is expressing poorly immunogenic antigens or is used in sensitive populations such as individuals who are immune suppressed, nutrient compromised, have an altered microbiome, or have an increased mucosal disease burden. Here, we review the current strategies being investigated to adjuvant the immune response to mucosal delivered LAB vaccine vectors. As these studies are reviewed, it is important to recognize that the adjuvant effect on the immune response may be altered by the mucosal route of administration (intranasal, oral, or intravaginal), genus and species of LAB used as the delivery vehicle, the antigen per se, and the mechanism of antigen display (secreted, surface-display, or intracellular). Careful study and selection of each of these variables will likely be necessary to develop optimized LAB mucosal vaccines.

## 2. Lactic Acid Bacteria Mechanisms of Immune Interaction and Activation

**Figure 1:**
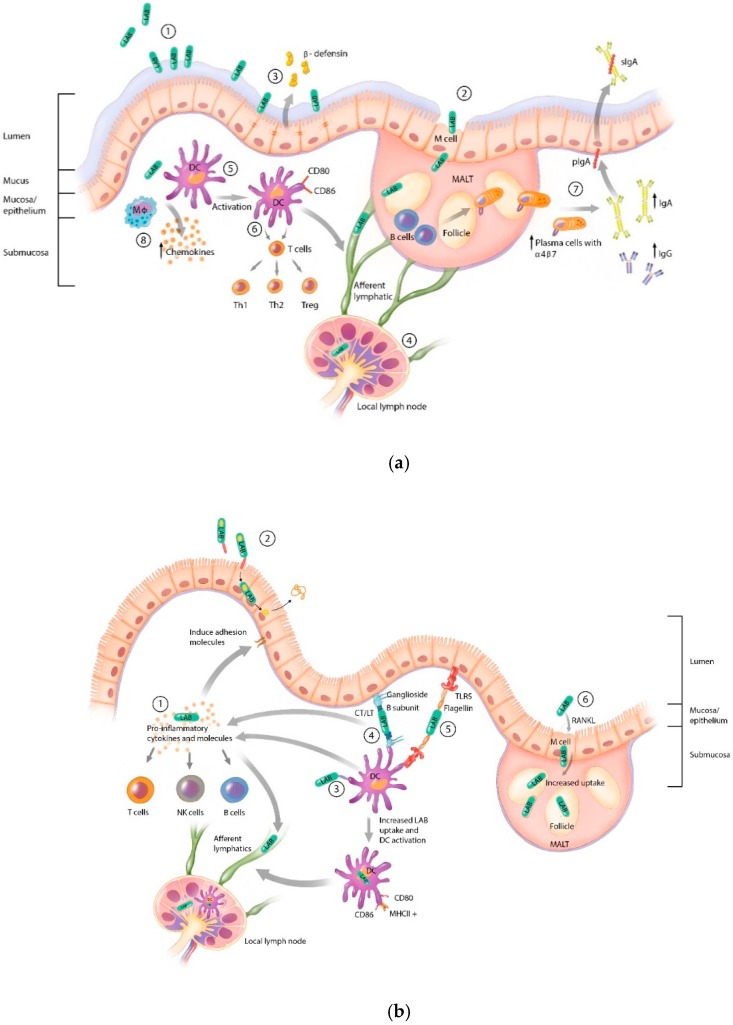

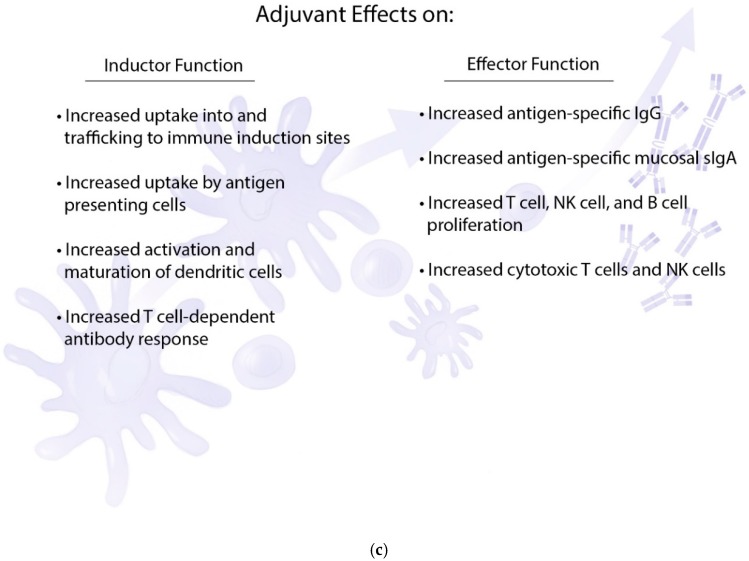
Lactic acid bacteria (LAB) interactions with the mucosa and mucosal immune system. (**a**) Endogenous LAB mucosal interactions. LAB possess the ability to bind to mucus (**1**), epithelial cells, and microfold (M) cells (**2**) allowing for uptake into mucosal associated lymphoid tissue (MALT) and trafficking to local lymph nodes (**4**) [24,25,26]. The interactions of LAB with the epithelium can induce epithelial defenses such as the secretion of β-defensin (**3**) [27,28]. LAB can activate macrophages (**8**) and dendritic cells (DCs) (**5**), which can traffic phagocytosed LAB to local immune induction sites (**4**) [29,30,31,32]. LAB also induce effector immune responses such as polarization of naïve T cells to Th1, Th2, and Treg cells (**6**) and humoral responses such as B cell proliferation, class switching to IgG and IgA, induction of long-lived plasma cells, and induction of the mucosal homing integrin α4β7 (**7**) [33,34]. (**b**) LAB mucosal adjuvant strategies. (**1**) LAB secretion of pro-inflammatory cytokines such as IL-12, IL-1β, and IL-2 activates T cells, NK cells, and B cells, induces epithelial cell adhesion molecule expression, and promotes trafficking of LAB to local lymph nodes. (**2**) LAB surface expression of the epithelial cell adhesion molecules InlA and/or FnBPA promotes binding and uptake of LAB by epithelial cells, delivery of eukaryotic expression plasmid, and secretion of protein. (**3**) LAB surface expression of DC-binding peptides results in targeting, increased uptake, and activation of DCs as well as trafficking to local immune induction sites. (**4**) Surface expression of LT or CT B subunit results in LAB binding to gangliosides on the surface of epithelial cells and DCs. Co-delivery of full toxins or CT/LT A subunit results in a pro-inflammatory response. (**5**) Surface-expressed flagellin, a TLR5 ligand, induces cytokine production by epithelial cells and direct activation of DCs. (**6**) LAB secretion of RANKL results in increased M cells and uptake of LAB into MALT. (**c**) Review of the effects of adjuvants on the immune response to LAB mucosal vaccination. LAB: Lactic acid bacteria; DC: Dendritic cell; Mϕ: Macrophage; MALT: Mucosal-associated lymphoid tissue; pIgA: Polymeric immunoglobulin receptor; sIgA: Secretory IgA; NK cells: Natural killer cells; M cells: Microfold cells; TLR: Toll-like receptor; RANKL: Receptor activator of nuclear factor kappa-B ligand; InlA: *Listeria monocytogenes* internalin A; FnBPA: Fibronectin-binding protein A; CT: Cholera toxin; LT: *E. coli* heat-labile toxin

To understand the effect that adjuvant strategies have on the immune response to a LAB mucosal vaccine, it is important to review the endogenous immune activating mechanisms possessed by LAB. A brief summary of typical LAB interactions with the mucosal immune system are depicted in Figure 1a.

Of note are the characteristics that make LAB especially attractive for use as a mucosal vaccine vector. LAB can stimulate innate immune response through the Gram-positive cell wall peptidoglycan and lipotechoic acid that activate the pattern-recognition receptors: Toll-like receptor (TLR) 2, nucleotide-binding oligomerization domain (NOD)-like receptor (NLR) family, and C-type lectin receptors [15,16,17,18]. Various species of LAB can also activate TLR3, TLR6, TLR9, and stimulate interferon responses [19,20,21]. Additionally, some LAB species can bind to intestinal mucus and the mucosal epithelium and/or microfold (M) cells resulting in mucosal colonization and increased uptake and transport into mucosal immune induction sites such as Peyer’s patches in the small intestine or tonsillar crypts. LAB can interact with antigen-presenting cells (APCs) such as dendritic cells (DC) and induce sIgA and IgG. The mechanism of DC activation and the resulting immune responses are highly dependent on the LAB strain. For example, it has been shown that murine DCs can have different responses depending on the strain of LAB and this is further complicated by the fact that these responses can be different even between DC subtypes [22,23]. This illustrates the complexity in selecting an appropriate LAB strain as a candidate vaccine vector. 

## 3. Mucosal Vaccine Adjuvant Strategies

Robust immune responses to mucosal vaccines have been difficult to achieve. In general, mucosal-delivered vaccines stimulate lower responses compared to systemic vaccines. To overcome this attenuated response, multiple mucosal adjuvants have been identified. Adjuvants of interest include: LAB expression of proteins that stimulate innate immune responses such as pathogen-associated molecular patterns (PAMPs), TLRs, NLRs, retinoic acid-inducible gene-like receptors (RLRs), and C-type lectins, targeting of professional APCs, immune modulating molecules (chemokines, cytokines), and bacterial toxins [35,36]. Molecular tools have been developed to allow for genetic manipulation of LAB making it possible to express adjuvants in multiple ways such as cell-surface display, secretion, and cytoplasmic [37]. The method of display should be carefully considered depending on the adjuvant, its mechanism of action, and the mode of LAB delivery. For instance, an adjuvant could be co-administered with a LAB vaccine that is delivered intranasally or intravaginally while an orally delivered LAB vaccine would encounter the harsh environment of the stomach, making co-administration inappropriate.

The majority of studies reviewed here used LAB to co-express antigen and adjuvant as opposed to co-administration of a separately produced adjuvant. This method of antigen/adjuvant LAB delivery is not only convenient but is also superior for oral delivery. LAB co-expression of antigen and adjuvant promotes survival of the adjuvant through the stomach and duodenum, enhances interaction with the mucosal surface including delivery to APCs and mucosal immune induction sites, and through colonization of the GI tract, prolonged delivery of the immune stimulating compound. Additionally, through their endogenous immune activation (Figure 1a), LAB can act in concert with the adjuvant to enhance immune responses.

The adjuvant strategies that have been employed with LAB vaccine vectors act through diverse mechanisms (Figure 1b) and evaluation of the adjuvant must be reviewed in the context of the route of delivery, specific LAB vector, and the expression strategy. These factors and the antigen and adjuvant employed, alterations in immune response, and protection against challenge are summarized in Section 4, Table 1, Table 2, Table 3, Table 4 and Table 5, and Figure 1b,c.

## 4. Lactic Acid Bacteria Adjuvant Strategies

### 4.1. Cytokine Secretion (Table 1)

Cytokines act to stimulate and attract immune cells. The selection of a cytokine for use as an adjuvant can be based on the desired immune response to vaccination and its known influence on immune cells. Three cytokines: IL-12, IL-1β, and IL-2 have been investigated for use as adjuvants with LAB vaccines. They have all been utilized as secreted molecules with the exception of one study by Li et al. where IL-12 was delivered as cDNA [38]. Cytokine expression strategies, as described below, have generally been successful and there are certainly other cytokines that could be explored. The challenge may be how to express the cytokine adjuvant in such a way that it does not add function to the bacterial vector and does not depend on antibiotic resistance to maintain expression from a plasmid. Cytokine expression could prove to be a challenge in the regulatory approval process.

#### 4.1.1. IL-12

The major sources of IL-12 are monocytes, macrophages, DCs, and neutrophils. The actions of this cytokine are to induce T cell and natural killer (NK) cell proliferation, increase IFN-γ, polarize CD4^+^ T cells to a Th1 phenotypes, and increase cytotoxicity [47]. LAB vaccines supplied with IL-12 have been used against viral induced neoplasia (human papilloma virus) and the intracellular pathogens *Leishmania major* and *Mycobacterium tuberculosis*. Immune responses were greater for the LAB administered with an IL-12 adjuvant as measured by IgG and sIgA (from bronchoalveolar lavage and intestinal wash). Additionally, there was elevated IFN-γ and IL-2 (to a lesser extent). IFN-γ polarizes T cells to a Th1 phenotype, important in responding to these intracellular pathogens, and IL-2 is important for T cell proliferation. This Th1 polarization is observed in other adjuvant studies reviewed here.

#### 4.1.2. IL-1β

IL-1β is secreted by monocytes and macrophages in response to TLR stimulation. It is secreted in an inactive form and cleaved by activated caspase-1 following assembly of the inflammasome [48]. Intracellular activation without secretion of IL-1β can also occur [49,50]. IL-1β is a pro-inflammatory cytokine and has been shown to act as a mucosal adjuvant [51]. It is important in T cell-mediated adaptive immune responses, induces adhesion molecules on mesenchymal and endothelial cells, and is an inducer of the B cell proliferation cytokine IL-6 [52,53]. The role of IL-1β on T cell-mediated antibody responses is important as T-dependent B cell responses often generate higher-affinity antibodies and increased memory. Secretion of IL-1β has been studied with both *L. casei* and *L. acidophilus*. In both, IL-1β increased IgG and mucosal sIgA when co-expressed with an antigen or delivered with an attenuated antigen (*Salmonella enterica*) [43,44]. Activated T cells and DCs resulted in increases in the inflammatory cytokines TNF-α, TNF-β, IL-6, and IL-4. The use of IL-1β as an adjuvant may have disadvantages as its pro-inflammatory effects may result in unintended consequences, although none were reported in the studies reviewed here.

#### 4.1.3. IL-2

IL-2 has been used as an adjuvant with *L. lactis* and *L. rhamnosus* GG. IL-2 plays a role in induction of immune responses, specifically proliferation and differentiation of CD4^+^ and CD8^+^ T cells, T regulatory (Treg) cells, and NK cells [54]. IL-2 also induces proliferation of intestinal epithelial cells at low concentrations while at high concentrations it can induce epithelial apoptosis. Secreted IL-2 resulted in increased IgG and sIgA and increased trafficking of LAB to mesenteric lymph nodes, an important site for sIgA induction [45,55]. While increased immune responses were observed using IL-2 as an adjuvant, altered levels of IL-2 have been found in inflammatory bowel disease patients and the complex interaction IL-2 has between inducing tolerance versus inflammation may be problematic for its use as a mucosal adjuvant [56,57,58].

### 4.2. Dendritic Cell (DC) Targeting Adjuvants (Table 2)

DCs are professional APCs critical for induction of adaptive immune responses and as such are enticing targets to enhance LAB immunogenicity. In the mucosa, DCs play a central role in inducing T and B cells and maintaining the balance of inflammation and tolerance. DCs take up antigens at mucosal surfaces in multiple ways. In the GI tract, DCs sample antigens through M cells or goblet cells, luminal sampling, binding to the neonatal Fc receptor, and apoptotic enterocytes [59]. The immune response generated by DCs depends on the method of antigen up-take and pro-inflammatory signals and can result in IgA class switching of B cells, increased sIgA, Th1 and cytotoxic lymphocyte induction, and induction of the mucosal homing integrin α4β7. Due to their importance in inducing mucosal immune responses, adjuvants that target DCs are attractive for use in mucosal delivered vaccines.

The most common method of targeting DCs with LAB is the surface expression of a DC-peptide attached to an antigen. 12-mer peptides were discovered through screening of a peptide phage display library for binding to the DC cell surface [60]. The peptides do not change the function of the DCs but target bound antigens for DCs resulting in the priming of T cells. This has been an active area of investigation with 10 publications evaluating peptide adjuvant qualities. In all these studies, the vaccines were delivered orally with the exception of one intranasal vaccine against avian influenza in chickens [61]. Delivery of LAB expressing antigen fused to a DC-peptide resulted in increased DC activation as determined by expression of MHCII, CD80, CD40, and CD86, increased serum IgG and mucosal sIgA, an increased Th1 T cell response, and protection from disease following challenge. DC-peptides seem to induce strong cell-mediated responses in addition to a robust antibody response. One study did report on possible tolerance induction with an increase in the Treg-associated cytokine TGF-β following vaccination and challenge with porcine epidemic diarrhea virus [62]. While TGF-β can be associated with Tregs, it can act in concert with IL-6 to induce Th17 cells. Thus, the significance of this finding is unknown, and more studies would be necessary to understand the mechanisms involved in this case.

Additional strategies have been reported for targeting of LAB mucosal vaccines to DCs, including surface expression of complement C3d3, anti-CD205, and the neonatal Fc receptor (FcRn) [63,64,65]. Of these three methods, only anti-CD205 acts solely by binding to DC cells. C3d3 can also target B cells and FcRn can bind to mucosal epithelial cells and other immune cells [66]. These approaches showed similar immune stimulating effects as compared to the DC-peptide adjuvant. Additionally, anti-CD205 was shown to be an effective adjuvant for delivery of a DNA plasmid to DCs and C3d3 acted to increase antibody responses and T and B cell proliferation to an intravaginal contraceptive vaccine. Taken together, DC targeting of LAB is a promising strategy that may also allow tuning of the immune outcome.

### 4.3. Secretion of Bacterial Toxins (Table 3)

Cholera toxin (CT) and the *E. coli* heat labile enterotoxin (LT) are well-studied mucosal adjuvants that have been used to enhance immune response to antigen delivered by LAB. CT activates DCs and promotes Th2 T cells and B cell isotype switching, while LT promotes antigen presentation and APC-T cell interactions [79]. The toxins are composed of two subunits: Active (A) and binding (B) [35,80]. The use of individual subunits is attractive as it can avoid the unwanted side effects associated with use of the whole toxin [81]. The specific mechanisms of cellular and immune system interaction are known for each subunit. The A subunit acts intracellularly to increase cAMP through ADP-ribosylating activity, and the B subunit binds to ganglioside on the surface of most cells. Importantly, the A subunit possesses the toxigenic effects but only when paired with the B subunit [82,83]. Meanwhile, the B subunit is generally considered non-toxic and enhances antigen-specific immune response through direct binding of immune cells and enhancement of antigen delivery. In the LAB studies reviewed here, CT and LT were delivered as full toxins co-administered with LAB or as individual subunits either surface-displayed or secreted.

CT and LT LAB adjuvants increased immune responses when compared to LAB mucosal (intranasal or oral) delivered vaccines alone. Outcomes included an increase in IgG and mucosal sIgA, increased protection against pathogen challenge, increased T cell responses (CD4^+^ and CD8^+^), and an increase in IFN-γ, IL-4, and IL-17. Of interest, studies utilizing CT subunits showed an immune response that was more Th1 polarized (increased IFN-γ) while studies using LT as an adjuvant resulted in both Th1 and Th2 responses (increased IFN-γ and IL-4) [84,85,86,87,88].

The use of CT and LT adjuvants is appealing due to the robust mucosal immune stimulating effects, but in vivo safety remains a serious concern. An example of the toxic effects of CT and LT was demonstrated by the intranasal influenza vaccine, Nasalflu. This vaccine showed increased immune response when delivered with whole LT and no toxicity was observed in clinical trials. Following approval, it was removed from the market after one year of clinical use due to increased incidence of facial paralysis [89]. It is possible that this unintended side effect could have been avoided with use of a single LT subunit or if administered through a different mucosal route (orally, for example). No toxicity was reported in the studies reviewed here but, regardless, further toxicity studies are necessary.

### 4.4. Bacterial Derived Adjuvants (Table 4)

Numerous bacterial proteins have been explored for use with LAB mucosal vaccines. These strategies take advantage of immune activating and invasive proteins that are utilized by pathogenic bacteria, and our considerable knowledge regarding host-bacteria interactions at the molecular level. In many cases the binding domains of bacterial proteins are well-characterized and relatively small, making incorporation of these peptides or short proteins easier to express in a LAB vaccine platform. This provides the opportunity to expand the PRR-activating repertoire and/or enhance interactions between the LAB construct and host.

#### 4.4.1. Toll-like Receptor (TLR) 5 Ligand

TLRs are expressed on many cell types and are an important activator of the innate immune response. TLR5 recognizes flagellin, a component of bacterial flagella, which stimulates production of chemokines and cytokines through myeloid differentiation factor 88 (MyD88) signaling [15]. In addition to TLR5 activation, flagellin binds to the cytosolic nucleotide binding oligomerization domain-like receptors (NLR) NLRC4, which leads to caspase-1 inflammasome activation [106]. There has been much interest in flagellin as a vaccine adjuvant due to its ease of expression, stability, and robust activation of immune response [35,107]. There is high expression of TLR5 in the lung, intestinal epithelial cells, monocytes/macrophages, and DCs. Due to this expression pattern, the use of flagellin as a mucosal adjuvant could result in immune activation as well as delivery of an antigen to APCs. Flagellin has been surface-expressed with multiple LAB including: *L. casei*, *L. gasseri*, and *L. acidophilus* [94,95,96]. Oral delivery of LAB expressing antigen and flagellin resulted in increased DC maturation, IgG and mucosal sIgA titers, and increases in both Th1 and Th2 cytokines. While the studies reviewed here only evaluated oral administration, flagellin could be a potent adjuvant for vaccines delivered through other mucosal routes. It has been shown to produce robust immune responses following intranasal delivery and TLR5 is expressed highly in numerous locations of the female reproductive tract, making it attractive for use with intravaginal delivered vaccines [108,109].

#### 4.4.2. Enterocyte Cell Targeting

Targeting LAB though surface expression of enterocyte binding proteins has been explored with the non-invasive LAB, *L. lactis*, through the use of *Listeria monocytogenes* internalin A (InIA) and/or *Staphylococcus aureus* fibronectin binding protein A (FnBPA) [97,98,99,100,101]. InlA is a cell wall protein that allows *L. monocytogenes* to bind and be internalized by epithelial cells [110]. FnBPA is also an epithelial cell binding protein that can bind to fibrinogen, elastin, and fibronectin allowing for internalization of *S. aureus* into non-phagocytic cells [111]. *L. lactis* with cell surface expression of InlA and/or FnBPA has been used to deliver DNA plasmids to intestinal epithelial cells. Delivery of β-lactoglobulin antigen DNA resulted in an increase of β-lactoglobulin within the intestinal lumen, increased Th1 and Th2 cytokine responses, and increased serum and bronchoalveolar fluid IgG and serum IgA (after intranasal delivery of DNA coding for *Mycobacterium tuberculosis* Ag85A) [98,99,100,101]. The use of InlA and FnBPA to deliver antigens to epithelial cells may be an effective mucosal vaccine strategy, especially if the desire is to deliver antigen via a eukaryotic expression plasmid (DNA vaccine).

#### 4.4.3. Additional Bacterial Derived Adjuvants

Other bacterial proteins and messengers have been explored as LAB adjuvants. These include: Muramyl dipeptide, *Neisseria meningitidis* PorA, c-di-AMP, and Salmonella resistance to complement killing [102,103,104,105]. Addition of these adjuvants to LAB mucosal vaccines resulted in an increased immune response and/or protection to challenge. The mechanism, if known, is described below.

Muramyl dipeptide (MDP) is a part of the bacterial cell wall and was delivered as a dipeptide with tuftsin, another biologically active compound. As mentioned above, LAB activate NOD2 and this is mediated through MDP breakdown products of the bacterial peptidoglycan. The exact mechanism of immune enhancement by MDP in combination with tuftsin is not fully elucidated but has been shown to activate APCs [112].

PorA is an outer membrane protein from the Gram-negative bacteria *Neisseria meningitidis*. This protein is immunodominant and, while using this protein as a vaccine antigen against *N. meningitidis* has not been successful, it has the potential to act as an adjuvant when conjugated to an antigen. For example, PorA increased the immune response to HpaA antigen from *Helicobacter pylori* [103]. The exact mechanism of action of PorA is still under investigation.

The bacterial second messenger c-di-AMP was evaluated as an intracytoplasmic adjuvant. c-di-AMP has numerous effects on the immune system including type I interferon responses, promotion of Th1 and Th2 responses, increased lymphocyte proliferation, and activation of APCs [113]. Delivery of c-di-AMP with an antigen against *Trypanosoma cruzi* resulted in a *T. cruzi*-specific immune response and is proof of concept that LAB can deliver biologically active c-di-AMP.

Finally, the use of Salmonella resistance to complement killing (RCK) protein was evaluated. This protein is important in interfering with complement killing and invasion into cells, including epithelial cells and APCs [114,115]. The use of RCK as a mucosal adjuvant was successful in increasing immune responses. The complete mechanism of immune activation is still unknown.

### 4.5. Other Adjuvant Strategies

There were three LAB adjuvant studies that did not fit into the above categories: Japanese herbal medicines (Juzen-taiho-to (JTT) and Hochi-ekki-to (HEY)), receptor activator of nuclear factor kappa-B ligand (RANKL), and thymosin α-1 [116,117,118]. They are briefly reviewed in Table 5 and their mechanisms of action described here.

The ability of the Japanese herbal medicines JTT and HEY to enhance immune response when co-administered with a *L. casei* oral human papilloma vaccine was evaluated [116]. These medicines have been shown to improve immune responses when delivered as an oral or intranasal adjuvant, but the exact mechanism of action is poorly described [119,120]. When delivered with *L. casei*, there was an increase in Th1 and Th2 cytokines. Other effects on the immune response following vaccination were not reported.

A study by Kim et al. aimed to increase the immune response to an oral *L. lactis* vaccine against the bacterium *Brachyspira hyodysenteriae* through the secretion of the M cell-inducing protein RANKL [117]. M cells are important for pathogen uptake from the intestinal lumen and transport into the Peyer’s patches [121]. *L. lactis* RANKL secretion increased M cell development, serum IgG, and fecal sIgA. This is an interesting adjuvant strategy as it acts through increased transport of the vaccine strain into Peyer’s patches and not through a pro-inflammatory or DC targeting method.

Surface-display of the immune-modifier peptide hormone, thymosin α-1, was evaluated as an adjuvant for an orally delivered *L. plantarum* vaccine against classical swine fever [118]. This peptide is secreted by the thymus and its use as a vaccine adjuvant has been shown to affect T cell maturation, cytotoxicity, Th1 and Th2 cytokine production, and increase antibody production [122,123]. Thymosin α-1 as a LAB adjuvant resulted in increased immune responses and protection from viral challenge in pigs.

## 5. Discussion

LAB have been investigated as potential mucosal vaccine platforms for nearly three decades [124,125]. Significant progress has been made to explore the inherent immunogenicity of various LAB, develop strategies to express recombinant proteins, and test antigen and adjuvant concepts [126]. To date, there is no licensed LAB-based vaccine primarily because necessary immunogenicity, efficacy, and durability have not been achieved. The desperate need for mucosal vaccine platforms continues, as does the promise of approaches that employ LAB. Success will depend on exploiting our current knowledge and emerging technologies. A thoughtful choice of LAB species and strain, antigens, and adjuvant will be required to generate immune protection in the target host. Adjuvants provide tremendous flexibility to direct the nature of the adaptive immune response by supplementing the inherent attributes of LAB. They can target the vaccine construct to a specific cell type, activate particular innate immune pathways, or be selected to drive a desired arm of the adaptive response.

Highly immunogenic mucosal adjuvants with appropriate safety profiles have been identified and here we reviewed many of these adjuvants in the context of a LAB vaccine vector [35,127]. LAB were able to produce and display or secrete these adjuvant cytokines, immune targeting peptides, bacterial toxins, and other immune stimulating bacterial proteins. Immune responses after mucosal administration were generally increased in all studies. Specific outcomes included: Increased humoral immune responses (increased IgG and sIgA), increased immune cell proliferation and activation, increased uptake of LAB into immune induction sites, and decreased morbidity and mortality following challenge with bacterial, viral, and parasitic pathogens. Additionally, these adjuvant strategies showed the ability to induce both Th1 and Th2 responses and increase sIgA titers at mucosal sites distant to the site of administration.

There were other interesting observations in the reviewed studies. The surface display of enterocyte-targeting bacterial proteins by *L. lactis* resulted in delivery of DNA plasmids to enterocytes and protein secretion into the intestinal lumen. This is a potential alternative strategy of protein antigen delivery and could also be utilized to deliver DNA to promote secretion of anti-viral or bacterial peptides [98,99,100,101]. Another reported benefit of these bacterial vectors is the ability to outcompete pathogens at mucosal surfaces. An example is a LAB vaccine against Enterotoxigenic *E. coli* (ETEC) with surface display of DC-peptide and ETEC fimbriae. The vector induced increased protective immune responses to ETEC infection and provided immediate protection from pathogen invasion by interfering with attachment of ETEC to intestinal cells [73].

As engineered LAB mucosal vaccines with enhanced immunogenicity are tested in vivo, further investigation is needed into the safety of these strategies. The addition of adjuvants to a vaccine should not cause long-lasting or debilitating local or systemic reactions or induce hypersensitivity reactions, autoimmunity, or neoplasia [128]. While LAB are regarded as safe and are used in numerous food products and health supplements, it is unknown if the inclusion of adjuvants would affect their safety profile. No adverse effects were reported in the studies reviewed here despite the use of CT and LT subunits or secretion of pro-inflammatory cytokines. Additionally, it is unknown if repeated exposure to genetically modified LAB would result in unintended immune responses as wild type probiotics are already known to induce and enhance mucosal antibody responses [129,130]. Whether off-target effects might result in anti-LAB (or other commensal) immune responses should be explored by analyzing the microbial community structure in vaccinated subjects.

## 6. Conclusions

The adjuvant strategies reviewed here are diverse and all resulted in increased immune responses. Next-generation LAB have the potential to be powerful mucosal vaccine vectors. Facile techniques that enable multiple genetic modifications, such as CRISPR/Cas, will likely usher in a new era of innovation that may enable the realization of a commercially viable LAB-based mucosal vaccine [37,131,132].

## Figures and Tables

**Table 1:** Cytokine adjuvant strategies for lactic acid bacteria.

Adjuvant	LAB	Expression	Antigen	Immune Response	Delivery	Species	Study
**IL-12**							
IL-12	*L. lactis*	Secreted	Human Papilloma Virus (E7)	Increased BAL IgG and sIgA	Intranasal	Murine C57BL/6	Bermudez-Humaran et al. 2005 [31]
				Increased IFN-γ CD4^+^ and 8^+^ T cells			
IL-12	*L. lactis* *L. plantarum*	Secreted	Human Papilloma Virus (E7)	*L. lactis*, Intranasal Delivery	Intranasal Oral	Murine C57BL/6	Cortes-Perez et al. 2007 [39]
				Increased Serum and GAL IgG; Increased GAL IgA			
				Increased IFN-γ			
				*L. plantarum*, Intranasal Delivery			
				Increased IFN-γ			
				Decreased Tumor Burden			
IL-12	*L. lactis*	Secreted	*Leishmania major Leishmania* (Homologue of Activated C Kinase)	Subcutaneous	Subcutaneous Oral	Murine BALB/c	Hugentobler et al. 2012 [40] Hugentobler et al. 2012 [41]
				Increased IgG1 and IgG2a			
				Increased IFN-γ			
				Decreased Parasite Load			
				Oral			
				Decreased Parasite Load			
				Increased Intestinal sIgA			
				Increased IFN-γ, IL-2			
IL-12	*L. lactis*	Cytoplasmic (DNA)	Human Papilloma Virus (E7)	Increased IFN-γ	Intranasal	Murine C57BL/6	Li et al. 2014 [38]
				Decreased Tumor Volume			
IL-12	*L. lactis* *L. plantarum*	Secreted (by *L. lactis* with *L. plantarum* Expressing the Antigen)	*Mycobacterium tuberculosis* (Subunit Epitopes: Ag85B, CFP-10, ESAT-6, Rv0475, Rv2031c)	Increased IgG	Oral	Murine BALB/c	Mustafa et al. 2018 [42]
				Increased IFN-γ, IL-2			
**IL-1** **β**							
IL-1β	*L. casei*	Secreted	*Salmonella enterica* (SE)	Increased IL-6, TNF-α, TGF-β	Oral	Murine C3H/HeJ	Kajikawa et al. 2010 [43]
				Increased IgG and Intestinal sIgA when Co-Delivered with SE			
IL-1β	*L. acidophilus*	Secreted	HIV-1 (Membrane Subunit Epitope)	Increased IgG, Intestinal and Vaginal sIgA	Oral	Murine BALB/c	Kajikawa et al. 2015 [44]
				Increased Intestinal and Vaginal Epitope-Specific IgA B cells			
				Increased IL-4			
**IL-2**							
IL-2	*L. rhamnosus* GG	Secreted	Green Florescent Protein (GFP)	Increased Trafficking to MLN and Spleen.	Oral	MurineC57BL/6 and BALB/c	Kandasamy et al. 2011 [45]
				Increased MLN T Cells, IgA B Cells, DCs			
				Increased GFP-Specific IgG and Fecal sIgA			
				Increased IFN-γ, IFN-α, IL-12			
IL-2	*L. lactis*	Secreted	Avian Influenza (Haemagglutinin 5)	Increased IgG and Serum IgA	Oral	Murine BALB/c	Szatraj et al. 2014 [46]

BAL: Bronchoalveolar lavage; GAL: Gastrointestinal lavage; MLN: Mesenteric lymph node.

**Table 2:** Dendritic cell (DC) adjuvant strategies for lactic acid bacteria.

Adjuvant	LAB	Expression	Antigen	Immune Response	Delivery	Species	Study
**DC-peptide**							
DC-pep	*L. acidophilus*	Surface-Display	*Bacillus anthracis* (Protective Antigen)	Increased IL-12, IL-10, TNFα, MCP-1	Oral	Murine A/J	Mohamadzadeh et al. 2009 [67]
				Increased Survival to Challenge			
DC-pep	*L. gasseri*	Surface-Display	*Bacillus anthracis* (Protective Antigen)	Increased IgG	Oral	Murine A/J	Mohamadzadeh et al. 2010 [68]
				Increased IL6, MCP-1, IFN-γ, IL-12			
				Increased Survival to Challenge			
				Increased T Cell Stimulation Following Challenge			
DC-pep	*L. plantarum*	Surface-Display	Newcastle Disease Virus (Hemagglutinin-Neuraminidase)	Increased Intestinal sIgA	Oral	Chicken	Jiang et al. 2015 [69]
				Increased Splenic and Peripheral Blood CD4^+^ T Cells			
				Increased Survival to Challenge			
DC-pep	*L. plantarum*	Surface-Display	Avian Influenza (Hemagglutinin)	Murine	Oral	Murine BALB/c Chicken	Shi et al. 2016 [70]
				Increased MLN and PP DC Activation (CD80^+^, CD86^+^) Increased IFN-γ Increased Survival to Challenge with Decreased Lung Viral Titer			
				Chicken			
				Increased CD3^+^ T Cell Proliferation and Increased CD3^+^CD4^+^/8^+^ PBMC Percentages Increased IFN-γ Increased BAL sIgA and Serum IgG Decreased Lung Viral Titer			
DC-pep	*L. plantarum*	Surface-Display	Avian influenza (Nucleoprotein and Matrix Protein)	Increased PP and LP DC Activation (CD80^+^, CD86^+^, CD40^+^, MHCII^+^)	Oral	Murine BALB/c, C57BL/6	Yang et al. 2016 [71]
				Increased PP IgA^+^ B Cells			
				Increased Fecal and BAL sIgA Titer			
				Increased IFN-γ, TNF-α			
				Increased T Cell Proliferation			
				Increased Survival Rate to Challenge and Decreased Lesions and Virus in Lung			
DC-pep	*L. casei*	Surface-Display	Porcine Epidemic Diarrhea Virus (Core Neutralizing Epitope)	Increased MLN and PP DC Activation (CD80^+^, CD86^+^, MHCII^+^)	Oral	Murine BALB/c	Wang et al. 2017 [72]
				Increased IgG, Viral Neutralization, and Genital Tract and Intestinal Mucus sIgA Titer			
				Increased Lymphocyte Proliferation			
				Increased IFN-γ, IL-4			
DC-pep	*L. plantarum*	Surface-Display	Enterotoxigenic *E. coli* (ETEC) (FaeG of K88 Fimbriae)	Increased Adhesion to Porcine Intestinal Cells and Decreased Attachment of ETEC (In Vitro)	Oral	Murine BALB/c	Yang et al. 2017 [73]
				Increased IgG and Intestinal sIgA			
				Increased Splenic and MLN B Cells and DCs			
				Increased TNF-α, IL-12, IL-4 Decreased Intestinal Lesions and Weight Loss Following Challenge			
DC-pep	*L. plantarum*	Surface-Display	Avian Influenza (Nucleoprotein and Matrix Protein)	Oral	Oral Intranasal	Chicken	Yang et al. 2017 [61]
				Increased Splenic CD4^+^ and CD8^+^ T Cells and T Cell Proliferation			
				Increased IgG and BAL sIgA			
				Decreased Disease and Lung Virus Intranasal			
				Increased Splenic CD8^+^ T Cells and T Cell Proliferation Increased BAL sIgA			
				Decreased Disease and Lung Virus			
DC-pep	*L. plantarum*	Surface-Display	*Eimeria tenella* (SO7)	Increased IgG and Intestinal sIgA	Oral	Chicken	Yang et al. 2017 [74]
				Decreased Oocyst Shedding and Cecum Lesion Scores Following Challenge			
DC-pep	*L. acidophilus*	Surface-Display	*Clostridium botulinum* (Botulinum Toxin Serotype A)	Approximately 70% Protection to Challenge (Protection B cell-Mediated)	Oral	Murine BALB/c	Sahay et al. 2018 [75]
DC-pep	*L. casei*	Surface-Display	Porcine Epidemic Diarrhea Virus (Collagenase-Digested Fragment of S Protein)	Increased IgG and Intestinal sIgA	Oral	Porcine	Hou et al. 2018 [62]
				Increased Th1/Th2 (IFN-γ/IL-4) CD4^+^ T Cells			
				Increased MLN TLR4, TLR9, and TGF-β and Decreased TNF-α Expression After Challenge			
				Increased Survival and Decreased Viral RNA After Challenge			
DC-pep	*L. plantarum*	Surface-Display	Porcine Epidemic Diarrhea Virus (S Protein)	Increased DC Activation (CD40/CD80^+^)	Oral	Murine BALB/c	Huang et al. 2018 [76]
				Increased PP IgA^+^ B Cells			
				Increased Serum IgG, Intestinal sIgA, and Neutralizing Antibodies (IgG/sIgA)			
				Increased MLN IFN-γ and IL-17			
DC-pep and M cell targeting peptide (Col)	*L. casei*	Surface-Display	Porcine Epidemic Diarrhea Virus (Core Neutralizing Epitope)	Increased IgG and Vaginal, Intestinal Mucus, and Fecal sIgA	Oral	Murine BALB/c	Ma et al. 2018 [77]
				Increased Splenic Lymphocyte Proliferation			
				Increased IFN-γ, IL-4			
				Increased Antibody-Mediated Virus Neutralization			
DC-pep	*L. casei*	Surface-Display	Bovine Viral Diarrhea Virus Glycoprotein E2	Increased PP DC Activation (CD40^+^)	Oral	Murine BALB/c	Wang et at. 2019 [78]
				Increased IgG and Intestinal sIgA			
				Increased Neutralizing IgG and sIgA			
				Increased IFN-γ, IL-4			
				Increased Splenic CD4^+^/CD8^+^ T Cells and T Cell Stimulation			
**Other**							
Complement (C3d3)	*L. casei*	Surface-Display	Human Chorionic Gonadotropin (hCG)	Increased Serum/Vaginal IgG and IgA with Increased Longevity of Response	Vaginal	Murine BALB/c and C57BL/6	Yao et al. 2007 [63]
				Increased T and B Cell Proliferation			
Anti-CD205	*L. plantarum*	Surface-Display	DNA (Plasmid)	Increased LAB DC Internalization	Oral	Murine BALB/c	Michon et al. 2015 [64]
				Increased Delivery of Plasmid to DCs			
Neonatal Fc receptor (FcRn)	*L. plantarum*	Surface-Display	Influenza (Ectodomain of Matrix 2 Protein)	Increased DC Activation (CD86^+^/CD80^+^)	Oral	Murine BALB/c	Yang et al. 2017 [65]
				Increased Splenic and MLN IFN-γ			
				Increased Intestinal sIgA			
				Increased MLN and PP IgA^+^ B cells			
				Increased Survival and Decreased Viral Load Following Challenge			

BAL: Bronchoalveolar lavage; PP: Peyer’s patch; MLN: Mesenteric lymph node; DC: Dendritic cell; LP: Lamina propria.

**Table 3:** Bacterial toxin adjuvant strategies for lactic acid bacteria.

Adjuvant	LAB	Expression	Antigen	Immune Response	Delivery	Species	Study
**Cholera Toxin (CT)**							
CT subunit B	*L. casei*	Co-administered	*Bordetella pertussis* (Filamentous Haemagglutinin Adhesin)	Increased IgG	Subcutaneous	Murine BALB/c	Colombi et al. 2006 [90]
CT subunit B	*L. lactis*	Co-administered	Avian Influenza (Hemagglutinin Antigen)	Increased IgG and Intestinal sIgA	Oral	Murine BALB/c	Lei et al. 2011 [84]
				Increased IFN-γ			
				Increased Survival to Challenge			
CT subunit B	*L. casei*	Secreted	None	Increased IgG	Intranasal	Murine BALB/c	Okuno et al. 2013 [91]
CT subunit A1	*L. casei*	Surface-Display	Influenza (Matrix Protein 2)	Increased IgG and BAL sIgA	Oral Intranasal	Murine BALB/c	Chowdhury et al. 2014 [85]
				Increased IFN-γ (Intranasal)			
				Increased Protection and Decreased Lung Viral Titer Following Challenge			
CT subunit A1	*L. casei*	Surface-Display	Influenza (Matrix Protein 2 and Hemagglutinin)	Increased IgG and BAL and Intestinal sIgA	Oral Intranasal	Murine BALB/c	Li et al. 2015 [86]
				Increased IFN-γ (Intranasal and Oral) and IL-4 (Intranasal)			
				Increased protection and decreased lung viral titer Following challenge			
				Longer Lasting Immune Response			
***E. coli* Heat-Liable Toxin (LT)**							
LT subunit B	*L. casei*	Surface-Display	Porcine rotavirus (VP4 capsid protein)	Increased Ocular, Vaginal, and Intestinal sIgA	Oral	Murine BALB/c	Qiao et al. 2009 [92]
LT subunit B	*L. casei*	Surface-Display Secreted	Porcine Epidemic Diarrhea Virus (Core Neutralizing Epitope)	Increased Intestinal, Vaginal, Nasal, Ocular, and Serum sIgA/IgA (Secreted Induced Highest Levels)	Oral	Murine BALB/c	Ge et al. 2012 [87]
				Increased Neutralizing Antibodies			
				Increased IFN-γ and IL-4			
LT subunit B and A (LTAK63)	*L. casei*	Surface-display	Enterotoxigenic *E. coli* (F4 (K88) fimbrial adhesion FaeG)	Increased IgG and Intestinal, Vaginal, and Nasal sIgA	Oral	Murine BALB/c	Yu et al. 2016 [93]
				Increased Splenic Lymphocyte Proliferation			
				Increased Protection to Challenge			
LT subunit B	*L. plantarum*	Surface-display	Avian influenza (hemagglutinin antigen)	Increased Intestinal sIgA	Oral	Murine BALB/c	Jiang et al. 2017 [88]
				Increased CD4^+^ T Cell IFN-γ (MLN), IL-4 (MLN, Splenic), IL-17 (MLN, Splenic) and CD8^+^ T Cell IFN-γ (MLN, Splenic)			
				Increased PP IgA^+^ B Cells			
				Increased Protection to Challenge			

BAL: Bronchoalveolar lavage; MLN: Mesenteric lymph node; PP: Peyer’s patch.

**Table 4:** Bacterial derived adjuvant strategies for lactic acid bacteria.

Adjuvant	LAB	Expression	Antigen	Immune Response	Delivery	Species	Study
**Toll-like receptor 5 ligand**							
Salmonella flagellin	*L. casei*	Surface-Display	*Salmonella enterica* (SipC)	Increased IL-8	Oral	Murine C3H/HeJ	Kajikawa et al. 2010 [94]
				Increased IgG			
				Increased IL-2, GM-CSF, IFN-γ			
Salmonella flagellin	*L. gasseri*	Surface-Display	None	Increased TLR5 Stimulation	Oral	Murine BALB/c	Stoeker et al. 2011 [95]
				Increased DC Maturation (MHCII^+^CD80^+^CD86^-^)			
				Increased IL17^+^ Lymphocytes			
				Increased Lamina Propria Plasma Cells			
Salmonella flagellin	*L. acidophilus*	Surface-Display	HIV-1 (Gag)	Increased IL-1β, IL-6	Oral	Murine BALB/c	Kajikawa et al. 2012 [96]
				Increased IgA-Secreting B Cells in FRT and LI			
				Decreased IFN-γ after HIV-1 In Vitro Exposure			
**Enterocyte targeting**							
*Listeria monocytogenes* Internalin A	*L. lactis*	Surface-Display	DNA (GFP)	Increased Entry into Epithelial Cells and Delivery of GFP Plasmid	Oral	Guinea pigs Hartley	Guimaraes et al. 2005 [97]
Internalin A	*L. lactis*	Surface-Display	DNA (β-Lactoglobulin Antigen)	Increased β-Lactoglobulin in Intestinal Lumen	Oral	Murine BALB/c	de Azevedo et al. 2012 [98]
Fibronectic-Binding Protein A	*L. lactis*	Surface-Display	DNA (β-Lactoglobulin Antigen)	Increased β-Lactoglobulin in Intestinal Lumen	Oral	Murine BALB/c	Pontes et al. 2012 [99]
Fibronectic-Binding Protein A and Internalin A	*L. lactis*	Surface-Display	DNA (β-Lactoglobulin Antigen)	Intranasal	Oral Intranasal	Murine BALB/c	Pontes et al. 2014 [100]
				Increased IL-4, IL-5, Decreased IFN-γ			
				Oral			
				Increased IL-5, Decreased IFN-γ			
Fibronectic-Binding Protein A	*L. lactis*	Surface-Display	DNA (*Mycobacterium tuberculosis* Ag85A)	Increased IFN-γ, TNF-α, IL-6	Intranasal	Murine C57BL/6	Mancha-Agresti et al. 2017 [101]
				Increased Serum IgG, IgA, and BAL IgG			
**Additional bacterial derived adjuvants**							
Muramyl Dipeptide and Tuftsin	*L. casei*	Secreted	Transmissible Gastroenteritis Virus (D Antigenic Site of the Spike Protein)	Increased Intestinal, Serum, Nasal, Ocular, and Vaginal sIgA	Oral	Murine BALB/c	Jiang et al. 2014 [102]
				Increased Splenic T Cell Proliferation			
				Increased Antibody-Mediated Viral Neutralization			
				Increased IL-10, TGF-β			
				Increased Th17 Cells and Decreased Treg Cells			
*Neisseria meningitidis* PorA	*L. lactis*	Cytoplasmic	Helicobacter pylori (HpaA)	Increased IgG	Oral	Murine BALB/c	Vasquez et al. 2015 [103]
c-di-AMP	*L. lactis*	Cytoplasmic	*Trypanosoma cruzi* (Trans-Sialidase Enzyme)	Increased Immune Response to *T. cruzi* Challenge	Oral	Murine BALB/c	Quintana et al. 2018 [104]
Salmonella Resistance to Complement Killing	*L. lactis*	Surface-display	Infectious Bursal Disease (VP2)	Increased Survival and Decreased Bursal Atrophy, Following Challenge (Intramuscular > Oral)	Oral Intramuscular	Chicken	Wang et al. 2019 [105]
				Increased Neutralizing Antibody (Intramuscular > Oral)			

DC: Dendritic cell; FRT: Female reproductive tract; LI: Large intestine; BAL: Bronchoalveolar lavage; TLR: Toll-like receptor.

**Table 5:** Other adjuvant strategies for lactic acid bacteria

Adjuvant	LAB	Expression	Antigen	Immune Response	Delivery	Species	Study
Herbal Medicine (JTT, HET)	*L. casei*	Co-administered	Human Papilloma Virus (E7)	Increased IFN-γ, IL-2 Secretion	Oral	Murine C57/BL6	Tagucki et al. 2012 [116]
RANKL	*L. lactis*	Secreted	*Brachyspira hyodysenteriae* (Membrane Protein B)	Increased M Cell Development	Oral	Murine BALB/c	Kim et al. 2015 [117]
				Increased IgG and Fecal sIgA			
Thymosin α-1	*L. plantarum*	Surface-Display	Classical Swine Fever (E2 Protein)	Increased IgG and Intestinal sIgA	Oral	Porcine	Xu et al. 2015 [118]
				Increased Virus Neutralizing Antibodies			
				Increased Cytotoxic Cells			
				Increased IFN-γ, IL-2, TNF-α			
				Increased Protection to Challenge			

RANKL: Receptor activator of nuclear factor kappa-B ligand; M cell: Microfold cell.

## References

[1] Markowiak P., Slizewska K. (2017). Effects of Probiotics, Prebiotics, and Synbiotics on Human Health. Nutrients.

[2] Gallo A., Passaro G., Gasbarrini A., Landolfi R., Montalto M. (2016). Modulation of microbiota as treatment for intestinal inflammatory disorders: An uptodate. World J. Gastroenterol..

[3] Boirivant M., Strober W. (2007). The mechanism of action of probiotics. Curr. Opin. Gastroenterol..

[4] Holmgren J., Czerkinsky C. (2005). Mucosal immunity and vaccines. Nat. Med..

[5] Neutra M.R., Kozlowski P.A. (2006). Mucosal vaccines: The promise and the challenge. Nat. Rev. Immunol..

[6] Kim S.H., Jang Y.S. (2017). The development of mucosal vaccines for both mucosal and systemic immune induction and the roles played by adjuvants. Clin. Exp. Vaccine Res..

[7] Zimmermann P., Curtis N. (2018). The influence of the intestinal microbiome on vaccine responses. Vaccine.

[8] Boyaka P.N. (2017). Inducing Mucosal IgA: A Challenge for Vaccine Adjuvants and Delivery Systems. J. Immunol..

[9] Zimmermann P., Curtis N. (2018). The influence of probiotics on vaccine responses—A systematic review. Vaccine.

[10] Wells J.M., Mercenier A. (2008). Mucosal delivery of therapeutic and prophylactic molecules using lactic acid bacteria. Nat. Rev. Microbiol..

[11] LeCureux J.S., Dean G.A. (2018). Lactobacillus Mucosal Vaccine Vectors: Immune Responses against Bacterial and Viral Antigens. mSphere.

[12] Rosales-Mendoza S., Angulo C., Meza B. (2016). Food-Grade Organisms as Vaccine Biofactories and Oral Delivery Vehicles. Trends Biotechnol..

[13] Gupta R.K., Siber G.R. (1995). Adjuvants for human vaccines--current status, problems and future prospects. Vaccine.

[14] Tregoning J.S., Russell R.F., Kinnear E. (2018). Adjuvanted influenza vaccines. Hum. Vaccin Immunother..

[15] Akira S., Uematsu S., Takeuchi O. (2006). Pathogen recognition and innate immunity. Cell.

[16] Girardin S.E., Boneca I.G., Viala J., Chamaillard M., Labigne A., Thomas G., Philpott D.J., Sansonetti P.J. (2003). Nod2 is a general sensor of peptidoglycan through muramyl dipeptide (MDP) detection. J. Biol. Chem..

[17] Smits H.H., Engering A., van der Kleij D., de Jong E.C., Schipper K., van Capel T.M., Zaat B.A., Yazdanbakhsh M., Wierenga E.A., van Kooyk Y. (2005). Selective probiotic bacteria induce IL-10-producing regulatory T cells in vitro by modulating dendritic cell function through dendritic cell-specific intercellular adhesion molecule 3-grabbing nonintegrin. J. Allergy Clin. Immunol..

[18] Konstantinov S.R., Smidt H., de Vos W.M., Bruijns S.C., Singh S.K., Valence F., Molle D., Lortal S., Altermann E., Klaenhammer T.R. (2008). S layer protein A of Lactobacillus acidophilus NCFM regulates immature dendritic cell and T cell functions. Proc. Natl. Acad. Sci. USA.

[19] Kawashima T., Ikari N., Watanabe Y., Kubota Y., Yoshio S., Kanto T., Motohashi S., Shimojo N., Tsuji N.M. (2018). Double-Stranded RNA Derived from Lactic Acid Bacteria Augments Th1 Immunity via Interferon-beta from Human Dendritic Cells. Front. Immunol..

[20] Ren Y., Pan H., Pan B., Bu W. (2016). Identification and functional characterization of three TLR signaling pathway genes in Cyclina sinensis. Fish Shellfish Immunol..

[21] Jounai K., Ikado K., Sugimura T., Ano Y., Braun J., Fujiwara D. (2012). Spherical lactic acid bacteria activate plasmacytoid dendritic cells immunomodulatory function via TLR9-dependent crosstalk with myeloid dendritic cells. PLoS ONE.

[22] Christensen H.R., Frokiaer H., Pestka J.J. (2002). Lactobacilli differentially modulate expression of cytokines and maturation surface markers in murine dendritic cells. J. Immunol..

[23] Hart A.L., Lammers K., Brigidi P., Vitali B., Rizzello F., Gionchetti P., Campieri M., Kamm M.A., Knight S.C., Stagg A.J. (2004). Modulation of human dendritic cell phenotype and function by probiotic bacteria. Gut.

[24] Lebeer S., Vanderleyden J., De Keersmaecker S.C. (2008). Genes and molecules of lactobacilli supporting probiotic action. Microbiol. Mol. Biol. Rev..

[25] Yanagihara S., Kanaya T., Fukuda S., Nakato G., Hanazato M., Wu X.R., Yamamoto N., Ohno H. (2017). Uromodulin-SlpA binding dictates Lactobacillus acidophilus uptake by intestinal epithelial M cells. Int. Immunol..

[26] Mercier-Bonin M., Chapot-Chartier M.P. (2017). Surface Proteins of Lactococcus lactis: Bacterial Resources for Muco-adhesion in the Gastrointestinal Tract. Front. Microbiol..

[27] Otte J.M., Podolsky D.K. (2004). Functional modulation of enterocytes by gram-positive and gram-negative microorganisms. Am. J. Physiol. Gastrointest. Liver Physiol..

[28] Schlee M., Harder J., Koten B., Stange E.F., Wehkamp J., Fellermann K. (2008). Probiotic lactobacilli and VSL#3 induce enterocyte beta-defensin 2. Clin. Exp. Immunol..

[29] Perdigon G., Maldonado Galdeano C., Valdez J.C., Medici M. (2002). Interaction of lactic acid bacteria with the gut immune system. Eur. J. Clin. Nutr..

[30] Yam K.K., Pouliot P., N’Diaye M.M., Fournier S., Olivier M., Cousineau B. (2008). Innate inflammatory responses to the Gram-positive bacterium Lactococcus lactis. Vaccine.

[31] Bermudez-Humaran L.G., Cortes-Perez N.G., Lefevre F., Guimaraes V., Rabot S., Alcocer-Gonzalez J.M., Gratadoux J.J., Rodriguez-Padilla C., Tamez-Guerra R.S., Corthier G. (2005). A novel mucosal vaccine based on live Lactococci expressing E7 antigen and IL-12 induces systemic and mucosal immune responses and protects mice against human papillomavirus type 16-induced tumors. J. Immunol..

[32] Kalina W.V., Mohamadzadeh M. (2005). Lactobacilli as natural enhancer of cellular immune response. Discov. Med..

[33] Bermudez-Humaran L.G. (2009). Lactococcus lactis as a live vector for mucosal delivery of therapeutic proteins. Hum. Vaccines.

[34] Bermudez-Humaran L.G., Kharrat P., Chatel J.M., Langella P. (2011). Lactococci and lactobacilli as mucosal delivery vectors for therapeutic proteins and DNA vaccines. Microb. Cell Fact..

[35] Rhee J.H., Lee S.E., Kim S.Y. (2012). Mucosal vaccine adjuvants update. Clin. Exp. Vaccine Res..

[36] Freytag L.C., Clements J.D. (2005). Mucosal adjuvants. Vaccine.

[37] Jiang B., Li Z., Ou B., Duan Q., Zhu G. (2019). Targeting ideal oral vaccine vectors based on probiotics: A systematical view. Appl. Microbiol. Biotechnol..

[38] Li Y., Li X., Liu H., Zhuang S., Yang J., Zhang F. (2014). Intranasal immunization with recombinant Lactococci carrying human papillomavirus E7 protein and mouse interleukin-12 DNA induces E7-specific antitumor effects in C57BL/6 mice. Oncol. Lett..

[39] Cortes-Perez N.G., Lefevre F., Corthier G., Adel-Patient K., Langella P., Bermudez-Humaran L.G. (2007). Influence of the route of immunization and the nature of the bacterial vector on immunogenicity of mucosal vaccines based on lactic acid bacteria. Vaccine.

[40] Hugentobler F., Di Roberto R.B., Gillard J., Cousineau B. (2012). Oral immunization using live Lactococcus lactis co-expressing LACK and IL-12 protects BALB/c mice against Leishmania major infection. Vaccine.

[41] Hugentobler F., Yam K.K., Gillard J., Mahbuba R., Olivier M., Cousineau B. (2012). Immunization against Leishmania major infection using LACK- and IL-12-expressing Lactococcus lactis induces delay in footpad swelling. PLoS ONE.

[42] Mustafa A.D., Kalyanasundram J., Sabidi S., Song A.A., Abdullah M., Abdul Rahim R., Yusoff K. (2018). Proof of concept in utilizing in-trans surface display system of Lactobacillus plantarum as mucosal tuberculosis vaccine via oral administration in mice. BMC Biotechnol..

[43] Kajikawa A., Masuda K., Katoh M., Igimi S. (2010). Adjuvant effects for oral immunization provided by recombinant Lactobacillus casei secreting biologically active murine interleukin-1{beta}. Clin. Vaccine Immunol..

[44] Kajikawa A., Zhang L., LaVoy A., Bumgardner S., Klaenhammer T.R., Dean G.A. (2015). Mucosal Immunogenicity of Genetically Modified Lactobacillus acidophilus Expressing an HIV-1 Epitope within the Surface Layer Protein. PLoS ONE.

[45] Kandasamy M., Selvakumari Jayasurya A., Moochhala S., Huat Bay B., Kun Lee Y., Mahendran R. (2011). Lactobacillus rhamnosus GG secreting an antigen and Interleukin-2 translocates across the gastrointestinal tract and induces an antigen specific immune response. Microbiol. Immunol..

[46] Szatraj K., Szczepankowska A.K., Saczynska V., Florys K., Gromadzka B., Lepek K., Plucienniczak G., Szewczyk B., Zagorski-Ostoja W., Bardowski J. (2014). Expression of avian influenza haemagglutinin (H5) and chicken interleukin 2 (chIL-2) under control of the ptcB promoter in Lactococcus lactis. Acta Biochim. Pol..

[47] Watford W.T., Moriguchi M., Morinobu A., O’Shea J.J. (2003). The biology of IL-12: Coordinating innate and adaptive immune responses. Cytokine Growth Factor Rev..

[48] Dinarello C.A. (2018). Overview of the IL-1 family in innate inflammation and acquired immunity. Immunol. Rev..

[49] Conos S.A., Lawlor K.E., Vaux D.L., Vince J.E., Lindqvist L.M. (2016). Cell death is not essential for caspase-1-mediated interleukin-1beta activation and secretion. Cell Death Differ..

[50] Boucher D., Monteleone M., Coll R.C., Chen K.W., Ross C.M., Teo J.L., Gomez G.A., Holley C.L., Bierschenk D., Stacey K.J. (2018). Caspase-1 self-cleavage is an intrinsic mechanism to terminate inflammasome activity. J. Exp. Med..

[51] Staats H.F., Ennis F.A. (1999). IL-1 is an effective adjuvant for mucosal and systemic immune responses when coadministered with protein immunogens. J. Immunol..

[52] Antoni G., Presentini R., Perin F., Tagliabue A., Ghiara P., Censini S., Volpini G., Villa L., Boraschi D. (1986). A short synthetic peptide fragment of human interleukin 1 with immunostimulatory but not inflammatory activity. J. Immunol..

[53] Shornick L.P., De Togni P., Mariathasan S., Goellner J., Strauss-Schoenberger J., Karr R.W., Ferguson T.A., Chaplin D.D. (1996). Mice deficient in IL-1beta manifest impaired contact hypersensitivity to trinitrochlorobenzone. J. Exp. Med..

[54] Abbas A.K., Trotta E., Simeonov D.R., Marson A., Bluestone J.A. (2018). Revisiting IL-2: Biology and therapeutic prospects. Sci. Immunol..

[55] Santiago A.F., Fernandes R.M., Santos B.P., Assis F.A., Oliveira R.P., Carvalho C.R., Faria A.M. (2008). Role of mesenteric lymph nodes and aging in secretory IgA production in mice. Cell. Immunol..

[56] Mishra J., Waters C.M., Kumar N. (2012). Molecular mechanism of interleukin-2-induced mucosal homeostasis. Am. J. Physiol. Cell Physiol..

[57] Brynskov J., Tvede N., Andersen C.B., Vilien M. (1992). Increased concentrations of interleukin 1 beta, interleukin-2, and soluble interleukin-2 receptors in endoscopical mucosal biopsy specimens with active inflammatory bowel disease. Gut.

[58] Pullman W.E., Doe W.F. (1992). IL-2 production by intestinal lamina propria cells in normal inflamed and cancer-bearing colons. Clin. Exp. Immunol..

[59] Chang S.Y., Ko H.J., Kweon M.N. (2014). Mucosal dendritic cells shape mucosal immunity. Exp. Mol. Med..

[60] Curiel T.J., Morris C., Brumlik M., Landry S.J., Finstad K., Nelson A., Joshi V., Hawkins C., Alarez X., Lackner A. (2004). Peptides identified through phage display direct immunogenic antigen to dendritic cells. J. Immunol..

[61] Yang W.T., Yang G.L., Shi S.H., Liu Y.Y., Huang H.B., Jiang Y.L., Wang J.Z., Shi C.W., Jing Y.B., Wang C.F. (2017). Protection of chickens against H9N2 avian influenza virus challenge with recombinant Lactobacillus plantarum expressing conserved antigens. Appl. Microbiol. Biotechnol..

[62] Hou X., Jiang X., Jiang Y., Tang L., Xu Y., Qiao X., Min L., Wen C., Ma G., Li Y. (2018). Oral Immunization against PEDV with Recombinant Lactobacillus casei Expressing Dendritic Cell-Targeting Peptide Fusing COE Protein of PEDV in Piglets. Viruses.

[63] Yao X.Y., Yuan M.M., Li D.J. (2007). Molecular adjuvant C3d3 improved the anti-hCGbeta humoral immune response in vaginal inoculation with live recombinant Lactobacillus expressing hCGbeta-C3d3 fusion protein. Vaccine.

[64] Michon C., Kuczkowska K., Langella P., Eijsink V.G., Mathiesen G., Chatel J.M. (2015). Surface display of an anti-DEC-205 single chain Fv fragment in Lactobacillus plantarum increases internalization and plasmid transfer to dendritic cells in vitro and in vivo. Microb. Cell Fact..

[65] Yang W.T., Yang G.L., Wang Q., Huang H.B., Jiang Y.L., Shi C.W., Wang J.Z., Huang K.Y., Jin Y.B., Wang C.F. (2017). Protective efficacy of Fc targeting conserved influenza virus M2e antigen expressed by Lactobacillus plantarum. Antivir. Res..

[66] Roopenian D.C., Akilesh S. (2007). FcRn: The neonatal Fc receptor comes of age. Nat. Rev. Immunol..

[67] Mohamadzadeh M., Duong T., Sandwick S.J., Hoover T., Klaenhammer T.R. (2009). Dendritic cell targeting of Bacillus anthracis protective antigen expressed by Lactobacillus acidophilus protects mice from lethal challenge. Proc. Natl. Acad. Sci. USA.

[68] Mohamadzadeh M., Durmaz E., Zadeh M., Pakanati K.C., Gramarossa M., Cohran V., Klaenhammer T.R. (2010). Targeted expression of anthrax protective antigen by Lactobacillus gasseri as an anthrax vaccine. Future Microbiol..

[69] Jiang Y., Hu J., Guo Y., Yang W., Ye L., Shi C., Liu Y., Yang G., Wang C. (2015). Construction and immunological evaluation of recombinant Lactobacillus plantarum expressing HN of Newcastle disease virus and DC- targeting peptide fusion protein. J. Biotechnol..

[70] Shi S.H., Yang W.T., Yang G.L., Zhang X.K., Liu Y.Y., Zhang L.J., Ye L.P., Hu J.T., Xing X., Qi C. (2016). Lactobacillus plantarum vaccine vector expressing hemagglutinin provides protection against H9N2 challenge infection. Virus Res..

[71] Yang W.T., Shi S.H., Yang G.L., Jiang Y.L., Zhao L., Li Y., Wang C.F. (2016). Cross-protective efficacy of dendritic cells targeting conserved influenza virus antigen expressed by Lactobacillus plantarum. Sci. Rep..

[72] Wang X., Wang L., Huang X., Ma S., Yu M., Shi W., Qiao X., Tang L., Xu Y., Li Y. (2017). Oral Delivery of Probiotics Expressing Dendritic Cell-Targeting Peptide Fused with Porcine Epidemic Diarrhea Virus COE Antigen: A Promising Vaccine Strategy against PEDV. Viruses.

[73] Yang G., Jiang Y., Tong P., Li C., Yang W., Hu J., Ye L., Gu W., Shi C., Shan B. (2017). Alleviation of enterotoxigenic Escherichia coli challenge by recombinant Lactobacillus plantarum expressing a FaeG- and DC-targeting peptide fusion protein. Benef. Microbes.

[74] Yang G., Yao J., Yang W., Jiang Y., Du J., Huang H., Gu W., Hu J., Ye L., Shi C. (2017). Construction and immunological evaluation of recombinant Lactobacillus plantarum expressing SO7 of Eimeria tenella fusion DC-targeting peptide. Vet. Parasitol..

[75] Sahay B., Colliou N., Zadeh M., Ge Y., Gong M., Owen J.L., Valletti M., Jobin C., Mohamadzadeh M. (2018). Dual-route targeted vaccine protects efficiently against botulinum neurotoxin A complex. Vaccine.

[76] Huang K.Y., Yang G.L., Jin Y.B., Liu J., Chen H.L., Wang P.B., Jiang Y.L., Shi C.W., Huang H.B., Wang J.Z. (2018). Construction and immunogenicity analysis of Lactobacillus plantarum expressing a porcine epidemic diarrhea virus S gene fused to a DC-targeting peptide. Virus Res..

[77] Ma S., Wang L., Huang X., Wang X., Chen S., Shi W., Qiao X., Jiang Y., Tang L., Xu Y. (2018). Oral recombinant Lactobacillus vaccine targeting the intestinal microfold cells and dendritic cells for delivering the core neutralizing epitope of porcine epidemic diarrhea virus. Microb. Cell Fact..

[78] Wang Y., Feng B., Niu C., Jia S., Sun C., Wang Z., Jiang Y., Cui W., Wang L., Xu Y. (2019). Dendritic Cell Targeting of Bovine Viral Diarrhea Virus E2 Protein Expressed by Lactobacillus casei Effectively Induces Antigen-Specific Immune Responses via Oral Vaccination. Viruses.

[79] Liang S., Hajishengallis G. (2010). Heat-labile enterotoxins as adjuvants or anti-inflammatory agents. Immunol. Investig.

[80] Petrovsky N. (2015). Comparative Safety of Vaccine Adjuvants: A Summary of Current Evidence and Future Needs. Drug Saf..

[81] Hajishengallis G., Arce S., Gockel C.M., Connell T.D., Russell M.W. (2005). Immunomodulation with enterotoxins for the generation of secretory immunity or tolerance: Applications for oral infections. J. Dent. Res..

[82] Agren L., Lowenadler B., Lycke N. (1998). A novel concept in mucosal adjuvanticity: The CTA1-DD adjuvant is a B cell-targeted fusion protein that incorporates the enzymatically active cholera toxin A1 subunit. Immunol. Cell Biol..

[83] Agren L., Sverremark E., Ekman L., Schon K., Lowenadler B., Fernandez C., Lycke N. (2000). The ADP-ribosylating CTA1-DD adjuvant enhances T cell-dependent and independent responses by direct action on B cells involving anti-apoptotic Bcl-2- and germinal center-promoting effects. J. Immunol..

[84] Lei H., Sheng Z., Ding Q., Chen J., Wei X., Lam D.M., Xu Y. (2011). Evaluation of oral immunization with recombinant avian influenza virus HA1 displayed on the Lactococcus lactis surface and combined with the mucosal adjuvant cholera toxin subunit B. Clin. Vaccine Immunol..

[85] Chowdhury M.Y., Li R., Kim J.H., Park M.E., Kim T.H., Pathinayake P., Weeratunga P., Song M.K., Son H.Y., Hong S.P. (2014). Mucosal vaccination with recombinant Lactobacillus casei-displayed CTA1-conjugated consensus matrix protein-2 (sM2) induces broad protection against divergent influenza subtypes in BALB/c mice. PLoS ONE.

[86] Li R., Chowdhury M.Y., Kim J.H., Kim T.H., Pathinayake P., Koo W.S., Park M.E., Yoon J.E., Roh J.B., Hong S.P. (2015). Mucosally administered Lactobacillus surface-displayed influenza antigens (sM2 and HA2) with cholera toxin subunit A1 (CTA1) Induce broadly protective immune responses against divergent influenza subtypes. Vet. Microbiol..

[87] Ge J.W., Liu D.Q., Li Y.J. (2012). Construction of recombinant lactobacilli expressing the core neutralizing epitope (COE) of porcine epidemic diarrhea virus and a fusion protein consisting of COE and Escherichia coli heat-labile enterotoxin B, and comparison of the immune responses by orogastric immunization. Can. J. Microbiol..

[88] Jiang Y., Yang G., Wang Q., Wang Z., Yang W., Gu W., Shi C., Wang J., Huang H., Wang C. (2017). Molecular mechanisms underlying protection against H9N2 influenza virus challenge in mice by recombinant Lactobacillus plantarum with surface displayed HA2-LTB. J. Biotechnol..

[89] Mutsch M., Zhou W., Rhodes P., Bopp M., Chen R.T., Linder T., Spyr C., Steffen R. (2004). Use of the inactivated intranasal influenza vaccine and the risk of Bell’s palsy in Switzerland. N. Engl. J. Med..

[90] Colombi D., Oliveira M.L., Campos I.B., Monedero V., Perez-Martinez G., Ho P.L. (2006). Haemagglutination induced by Bordetella pertussis filamentous haemagglutinin adhesin (FHA) is inhibited by antibodies produced against FHA(430-873) fragment expressed in Lactobacillus casei. Curr. Microbiol..

[91] Okuno T., Kashige N., Satho T., Irie K., Hiramatsu Y., Sharmin T., Fukumitsu Y., Uyeda S., Yamada S., Harakuni T. (2013). Expression and secretion of cholera toxin B subunit in lactobacilli. Biol. Pharm. Bull..

[92] Qiao X., Li G., Wang X., Li X., Liu M., Li Y. (2009). Recombinant porcine rotavirus VP4 and VP4-LTB expressed in Lactobacillus casei induced mucosal and systemic antibody responses in mice. BMC Microbiol..

[93] Yu M., Qi R., Chen C., Yin J., Ma S., Shi W., Wu Y., Ge J., Jiang Y., Tang L. (2017). Immunogenicity of recombinant Lactobacillus casei-expressing F4 (K88) fimbrial adhesin FaeG in conjunction with a heat-labile enterotoxin A (LTAK63) and heat-labile enterotoxin B (LTB) of enterotoxigenic Escherichia coli as an oral adjuvant in mice. J. Appl. Microbiol..

[94] Kajikawa A., Igimi S. (2010). Innate and acquired immune responses induced by recombinant Lactobacillus casei displaying flagellin-fusion antigen on the cell-surface. Vaccine.

[95] Stoeker L., Nordone S., Gunderson S., Zhang L., Kajikawa A., LaVoy A., Miller M., Klaenhammer T.R., Dean G.A. (2011). Assessment of Lactobacillus gasseri as a candidate oral vaccine vector. Clin. Vaccine Immunol..

[96] Kajikawa A., Zhang L., Long J., Nordone S., Stoeker L., LaVoy A., Bumgardner S., Klaenhammer T., Dean G. (2012). Construction and immunological evaluation of dual cell surface display of HIV-1 gag and Salmonella enterica serovar Typhimurium FliC in Lactobacillus acidophilus for vaccine delivery. Clin. Vaccine Immunol..

[97] Guimaraes V.D., Gabriel J.E., Lefevre F., Cabanes D., Gruss A., Cossart P., Azevedo V., Langella P. (2005). Internalin-expressing Lactococcus lactis is able to invade small intestine of guinea pigs and deliver DNA into mammalian epithelial cells. Microbes Infect..

[98] de Azevedo M., Karczewski J., Lefevre F., Azevedo V., Miyoshi A., Wells J.M., Langella P., Chatel J.M. (2012). In vitro and in vivo characterization of DNA delivery using recombinant Lactococcus lactis expressing a mutated form of L. monocytogenes Internalin, A. BMC Microbiol..

[99] Pontes D., Innocentin S., Del Carmen S., Almeida J.F., Leblanc J.G., de Moreno de Leblanc A., Blugeon S., Cherbuy C., Lefevre F., Azevedo V. (2012). Production of Fibronectin Binding Protein A at the surface of Lactococcus lactis increases plasmid transfer in vitro and in vivo. PLoS ONE.

[100] Pontes D., Azevedo M., Innocentin S., Blugeon S., Lefevre F., Azevedo V., Miyoshi A., Courtin P., Chapot-Chartier M.P., Langella P. (2014). Immune response elicited by DNA vaccination using Lactococcus lactis is modified by the production of surface exposed pathogenic protein. PLoS ONE.

[101] Mancha-Agresti P., de Castro C.P., Dos Santos J.S.C., Araujo M.A., Pereira V.B., LeBlanc J.G., Leclercq S.Y., Azevedo V. (2017). Recombinant Invasive Lactococcus lactis Carrying a DNA Vaccine Coding the Ag85A Antigen Increases INF-gamma, IL-6, and TNF-alpha Cytokines after Intranasal Immunization. Front. Microbiol..

[102] Jiang X., Yu M., Qiao X., Liu M., Tang L., Jiang Y., Cui W., Li Y. (2014). Up-regulation of MDP and tuftsin gene expression in Th1 and Th17 cells as an adjuvant for an oral Lactobacillus casei vaccine against anti-transmissible gastroenteritis virus. Appl. Microbiol. Biotechnol..

[103] Vasquez A.E., Manzo R.A., Soto D.A., Barrientos M.J., Maldonado A.E., Mosqueira M., Avila A., Touma J., Bruce E., Harris P.R. (2015). Oral administration of recombinant Neisseria meningitidis PorA genetically fused to H. pylori HpaA antigen increases antibody levels in mouse serum, suggesting that PorA behaves as a putative adjuvant. Hum. Vaccin Immunother..

[104] Quintana I., Espariz M., Villar S.R., Gonzalez F.B., Pacini M.F., Cabrera G., Bontempi I., Prochetto E., Stulke J., Perez A.R. (2018). Genetic Engineering of Lactococcus lactis Co-producing Antigen and the Mucosal Adjuvant 3’ 5’- cyclic di Adenosine Monophosphate (c-di-AMP) as a Design Strategy to Develop a Mucosal Vaccine Prototype. Front. Microbiol..

[105] Wang W., Song Y., Liu L., Zhang Y., Wang T., Zhang W., Li K., Qi X., Gao Y., Gao L. (2019). Neutralizing-antibody-mediated protection of chickens against infectious bursal disease via one-time vaccination with inactivated recombinant Lactococcus lactis expressing a fusion protein constructed from the RCK protein of Salmonella enterica and VP2 of infectious bursal disease virus. Microb. Cell Fact..

[106] Miao E.A., Alpuche-Aranda C.M., Dors M., Clark A.E., Bader M.W., Miller S.I., Aderem A. (2006). Cytoplasmic flagellin activates caspase-1 and secretion of interleukin 1beta via Ipaf. Nat. Immunol..

[107] Cui B., Liu X., Fang Y., Zhou P., Zhang Y., Wang Y. (2018). Flagellin as a vaccine adjuvant. Expert Rev. Vaccines.

[108] Hong S.H., Byun Y.H., Nguyen C.T., Kim S.Y., Seong B.L., Park S., Woo G.J., Yoon Y., Koh J.T., Fujihashi K. (2012). Intranasal administration of a flagellin-adjuvanted inactivated influenza vaccine enhances mucosal immune responses to protect mice against lethal infection. Vaccine.

[109] Fazeli A., Bruce C., Anumba D.O. (2005). Characterization of Toll-like receptors in the female reproductive tract in humans. Hum. Reprod..

[110] Gaillard J.L., Berche P., Frehel C., Gouin E., Cossart P. (1991). Entry of L. monocytogenes into cells is mediated by internalin, a repeat protein reminiscent of surface antigens from gram-positive cocci. Cell.

[111] Innocentin S., Guimaraes V., Miyoshi A., Azevedo V., Langella P., Chatel J.M., Lefevre F. (2009). Lactococcus lactis expressing either Staphylococcus aureus fibronectin-binding protein A or Listeria monocytogenes internalin A can efficiently internalize and deliver DNA in human epithelial cells. Appl. Environ. Microbiol..

[112] Wardowska A., Dzierzbicka K., Menderska A., Trzonkowski P. (2013). New conjugates of tuftsin and muramyl dipeptide as stimulators of human monocyte-derived dendritic cells. Protein Pept. Lett..

[113] Skrnjug I., Rueckert C., Libanova R., Lienenklaus S., Weiss S., Guzman C.A. (2014). The mucosal adjuvant cyclic di-AMP exerts immune stimulatory effects on dendritic cells and macrophages. PLoS ONE.

[114] Heffernan E.J., Reed S., Hackett J., Fierer J., Roudier C., Guiney D. (1992). Mechanism of resistance to complement-mediated killing of bacteria encoded by the Salmonella typhimurium virulence plasmid gene rck. J. Clin. Investig..

[115] Rosselin M., Virlogeux-Payant I., Roy C., Bottreau E., Sizaret P.Y., Mijouin L., Germon P., Caron E., Velge P., Wiedemann A. (2010). Rck of Salmonella enterica, subspecies enterica serovar enteritidis, mediates zipper-like internalization. Cell Res..

[116] Taguchi A., Kawana K., Yokoyama T., Adachi K., Yamashita A., Tomio K., Kojima S., Oda K., Fujii T., Kozuma S. (2012). Adjuvant effect of Japanese herbal medicines on the mucosal type 1 immune responses to human papillomavirus (HPV) E7 in mice immunized orally with Lactobacillus-based therapeutic HPV vaccine in a synergistic manner. Vaccine.

[117] Kim J.I., Park T.E., Maharjan S., Li H.S., Lee H.B., Kim I.S., Piao D., Lee J.Y., Cho C.S., Bok J.D. (2015). Soluble RANKL expression in Lactococcus lactis and investigation of its potential as an oral vaccine adjuvant. BMC Immunol..

[118] Xu Y.G., Guan X.T., Liu Z.M., Tian C.Y., Cui L.C. (2015). Immunogenicity in Swine of Orally Administered Recombinant Lactobacillus plantarum Expressing Classical Swine Fever Virus E2 Protein in Conjunction with Thymosin alpha-1 as an Adjuvant. Appl. Environ. Microbiol..

[119] Underwood J.R., Chivers M., Dang T.T., Licciardi P.V. (2009). Stimulation of tetanus toxoid-specific immune responses by a traditional Chinese herbal medicine. Vaccine.

[120] Kiyohara H., Nagai T., Munakata K., Nonaka K., Hanawa T., Kim S.J., Yamada H. (2006). Stimulating effect of Japanese herbal (kampo) medicine, hochuekkito on upper respiratory mucosal immune system. Evid. Based Complement. Altern. Med..

[121] Wang M., Gao Z., Zhang Z., Pan L., Zhang Y. (2014). Roles of M cells in infection and mucosal vaccines. Hum. Vaccin Immunother..

[122] Jiang Y.F., Ma Z.H., Zhao P.W., Pan Y., Liu Y.Y., Feng J.Y., Niu J.Q. (2010). Effect of thymosin-alpha(1) on T-helper 1 cell and T-helper 2 cell cytokine synthesis in patients with hepatitis B virus e antigen-positive chronic hepatitis B. J. Int. Med. Res..

[123] Li C.L., Zhang T., Saibara T., Nemoto Y., Ono M., Akisawa N., Iwasaki S., Maeda T., Onishi S. (2002). Thymosin alpha1 accelerates restoration of T cell-mediated neutralizing antibody response in immunocompromised hosts. Int. Immunopharmacol..

[124] Gerritse K., Posno M., Schellekens M.M., Boersma W.J., Claassen E. (1990). Oral administration of TNP-Lactobacillus conjugates in mice: A model for evaluation of mucosal and systemic immune responses and memory formation elicited by transformed lactobacilli. Res. Microbiol..

[125] Marteau P., Rambaud J.C. (1993). Potential of using lactic acid bacteria for therapy and immunomodulation in man. FEMS Microbiol. Rev..

[126] Takahashi K., Orito N., Tokunoh N., Inoue N. (2019). Current issues regarding the application of recombinant lactic acid bacteria to mucosal vaccine carriers. Appl. Microbiol. Biotechnol..

[127] Apostolico Jde S., Lunardelli V.A., Coirada F.C., Boscardin S.B., Rosa D.S. (2016). Adjuvants: Classification, Modus Operandi, and Licensing. J. Immunol. Res..

[128] Edelman R. (1980). Vaccine adjuvants. Rev. Infect. Dis..

[129] Umesaki Y., Setoyama H. (2000). Structure of the intestinal flora responsible for development of the gut immune system in a rodent model. Microbes Infect..

[130] Hardy H., Harris J., Lyon E., Beal J., Foey A.D. (2013). Probiotics, prebiotics and immunomodulation of gut mucosal defences: Homeostasis and immunopathology. Nutrients.

[131] Stout E., Klaenhammer T., Barrangou R. (2017). CRISPR-Cas Technologies and Applications in Food Bacteria. Annu. Rev. Food Sci. Technol..

[132] van Pijkeren J.P., Barrangou R. (2017). Genome Editing of Food-Grade Lactobacilli to Develop Therapeutic Probiotics. Microbiol. Spectr..

